# Use of a multilocus variable-number tandem repeat analysis method for molecular subtyping and phylogenetic analysis of *Neisseria meningitidis *isolates

**DOI:** 10.1186/1471-2180-6-44

**Published:** 2006-05-11

**Authors:** Jui-Cheng Liao, Chun-Chin Li, Chien-Shun Chiou

**Affiliations:** 1 The Third Branch Office, Center for Disease Control, Taichung 408, Taiwan

## Abstract

**Background:**

The multilocus variable-number tandem repeat (VNTR) analysis (MLVA) technique has been developed for fine typing of many bacterial species. The genomic sequences of *Neisseria meningitidis *strains Z2491, MC58 and FAM18 have been available for searching potential VNTR loci by computer software. In this study, we developed and evaluated a MLVA method for molecular subtyping and phylogenetic analysis of *N. meningitidis *strains.

**Results:**

A total of 12 VNTR loci were identified for subtyping and phylogenetic analysis of 100 *N. meningitidis *isolates, which had previously been characterized by pulsed-field gel electrophoresis (PFGE) and multilocus sequence typing. The number of alleles ranges from 3 to 40 for the 12 VNTR loci; theoretically, the numbers of alleles can generate more than 5 × 10^11 ^MLVA types. In total, 93 MLVA types were identified in the 100 isolates, indicating that MLVA is powerful in discriminating *N. meningitidis *strains. In phylogenetic analysis with the minimal spanning tree method, clonal relationships, established with MLVA types, agreed well with those built with ST types.

**Conclusion:**

Our study indicates that the MLVA method has a higher degree of resolution than PFGE in discriminating *N. meningitidis *isolates and may be a useful tool for phylogenetic studies of strains evolving over different time scales.

## Background

*Neisseria meningitidis *is one of the major causative agents of bacterial meningitis and septicemia in children and young adults [1]. Periodically, it causes large epidemics in Africa, especially in the sub-Saharan meningitis belt, and in Asia [1]; however, it is still a serious problem in many industrialized countries [2,3]. Occasionally, a meningococcal pandemic occurs after large population movements, such as pilgrimages [4,5].

Epidemiological studies of *N. meningitidis*, using various subtyping methods, allow the identification of a disease outbreak and investigation of the disseminating meningococcal strains. With the advent of molecular biology, a number of molecular methods have been developed for epidemiological studies of *N. meningitidis*. Among the methods, pulsed-field gel electrophoresis (PFGE) and multilocus sequence typing (MLST) are the most frequently used subtyping techniques [6,7]. PFGE usually exhibits high discrimination for bacterial isolates, but it generates fingerprint image data that makes a comparison between laboratories difficult. In contrast, MLST is based on sequence data from seven conserved housekeeping genes; sequences that differ at even a single nucleotide are assigned to different alleles. The combination of alleles at the seven housekeeping genes is designated the sequence type (ST) of the isolate; numerous STs can be obtained. A *Neisseria *MLST database has been established that allows STs to be compared electronically via the Internet. STs are grouped into clonal complexes by their similarity to a central allelic profile (genotype). These central genotypes are identified by a number of heuristic means, including BURST and split decomposition, along with feedback from public health laboratories and epidemiologists. Once a central genotype has been identified, clonal complexes are defined as including any ST that matches the central genotype at four or more loci unless it more closely matches another central genotype [8]. The accumulation of nucleotide changes in housekeeping genes is a relatively slow process, and the allelic profile of a meningococcal strain is stable over time. Therefore, MLST is a powerful tool for study of global epidemiology of meningococci [6]. However, MLST provides lower discrimination than PFGE for fine typing of some clonal groups of *N. meningitidis *[9].

In recent years, the multilocus variable-number tandem repeat (VNTR) analysis (MLVA) technique has been developed for fine typing of many bacterial species [10-19]]. In addition, Yazdankhah et al. [20] have recently developed a MLVA method with four VNTR loci for genotyping of *N. meningitidis *isolates and successfully differentiated the serogroup W135 isolates from sporadic cases and outbreaks. In this study, we successfully developed a MLVA method with 12 VNTR loci to analyze a panel of *N. meningitidis *isolates, which had previously been characterized by PFGE and MLST.

## Results

### Identification of potential VNTR loci

Initially, 23 potential VNTR loci with short lengths of repeat units (≦ 30 bp) were selected from a list of repeat loci identified by the VNTRDB program at the three genomes of *N. meningitidis *strains Z2491, MC58 and FAM18. After evaluation with 10 genetically distinct *N. meningitidis *strains, 12 VNTR loci were then chosen for genotyping 100 *N. meningitidis *isolates. The remaining 11 VNTR loci were abandoned because multiple bands were produced or no PCR products were detected in all the 10 isolates. Four of the 11 loci were *opa *genes, which existed in multiple copies with various repeat numbers in *Neisseria *spp. [21]. Such loci were too complicated to be useful for MLVA genotyping. Among the 12 loci, three (NMTR1, NMTR9, NMTR12) have been characterized by Yazdankhah et al. [20]. Both VNTR06 and VNTR08 loci, described by Yazdankhah et al. [20], are actually the same locus equivalent to the NMTR9 locus described in this study. In the genomic sequence of *N. meningitidis *strain MC58, the primers VNTR06-F and VNTR06-R are at positions 286076–286057 and 285626–285649, and VNRT08-F and VNTR08-R at 285707–285726 and 286018–285999, respectively. Both primer sets amplify the same VNTR locus. The lengths of repeat units for the 12 repeat loci ranged from 4 to 30 bp, 7 of the 12 loci were multiples of 3 bp. NMTR12 is a compound tandem repeat with 12- and 13-bp repeat units arranging in variable numbers and sequences. This compound tandem repeat was verified by sequencing all the amplicons from 100 *N. meningitidis *isolates. Of the 12 loci, at least 9 were located in coding region of annotated genes (Table 1). The VNTRDB program used each of the three genomic sequences in turn as a "parent" sequence to search repeat loci and, then, located each of the loci at the other two genomes, so that a locus, for example NMTR9a with only one repeat unit in strains MC58 but with 2 repeat units in strain Z2491 and 3 repeat units in strain FAM18, could be found (Table 1).

### MLVA genotyping

The MLVA genotyping was performed on 100 *N. meningitidis *isolates, which were collected between 1996 and 2002, and their PFGE patterns and ST types were characterized previously [9]. The results showed that the majority of the isolates carried only one copy of each of the 12 loci; however, five isolates carried extra copy of NMTR1, NMTR7, NMTR9 or NMTR18 locus, two isolates did not carry the NMTR1 locus, and three isolates did not carry the NMTR12 locus (Table 2). The number of alleles at each of the 12 loci ranged from 3 to 40 alleles counted on the 100 isolates analyzed (Table 3). Six loci (NMTR1, NMTR2, NMTR7, NMTR9, NMTR10 and NMTR12) had more than 10 alleles and four loci (NMTR1, NMTR2, NMTR7 and NMTR9) had a high allelic polymorphism index (≥ 0.9) (Table 3). Based on the allele number for each of the 12 loci determined in this study, at least 5 × 10^11 ^MLVA allelic profiles (MLVA types) are expected.

A total of 93 MLVA types were identified for the 100 isolates (Table 2). The majority of MLVA types represented only one isolate; however, each TW4, TW5, TW51, TW52, and TW62 types represented two isolates and TW3 represented three isolates. TW62 was identified in two serogroup B isolates (NM255 and NM256), which were obtained from two cases in a meningococcal disease outbreak in a family. TW52 was identified in two serogroup C isolates (NM377 and NM378) with a close epidemiological relationship. TW3, TW4, and TW5 were identified in serogroup Y isolates collected from sporadic cases; the isolates were derived from a newly imported clone [9]. The two serogroup W135 isolates with TW51 type were collected in cases at a 2-year interval.

As shown in the previous study [9], PFGE exhibited a higher degree of discrimination than MLST for the isolates analyzed. However, the results of this study showed that MLVA exhibited much higher resolution than PFGE on the same panel of isolates. MLVA discriminated all of the serogroup B isolates and 29 of 31 serogroup W135 isolates, which were collected from sporadic cases (Table 2). In contrast, only two ST type and four PFGE patterns were identified in the 31 serogroup W135 isolates (Table 2). Only one ST type and two PFGE patterns were identified in the 11 serogroup Y isolates (Table 2). However, these isolates were further discriminated into seven MLVA genotypes.

### Phylogenetic analysis

**Figure 1:**
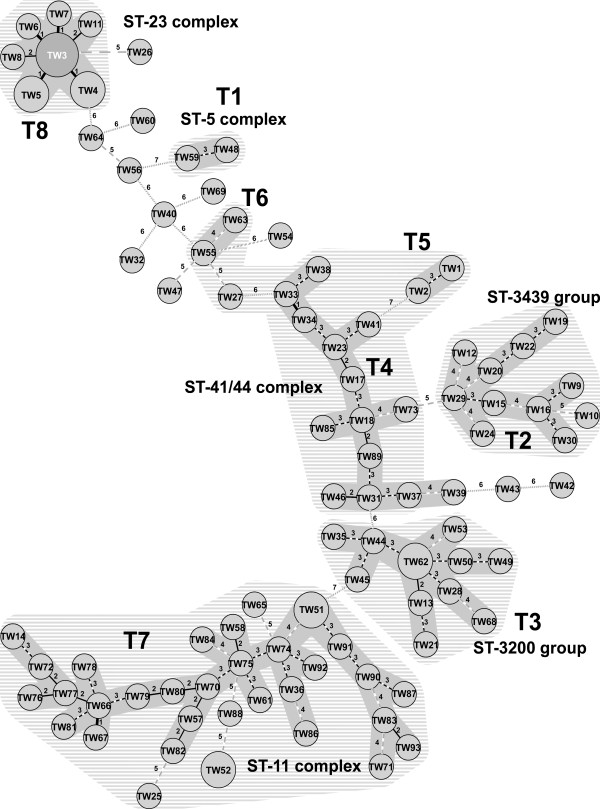
Phylogenetic tree built with MLVA profiles. Minimum Spanning Tree diagram, consisting of 93 MLVA types are identified. Differences in loci between two MLVA types are numbered. Circle size is proportional to the number of isolates belonging to an MLVA type. Two or more MLVA types differing in four or less loci are regarded as a group. MLVA types in groups (T1 – T8) are marked in dark gray shadow and ST groups or ST complexes marked in light dashed lines.

The clonal relationships among the 100 isolates were constructed with the MLVA types by the minimal spanning tree (MST) method. In the analysis with 12 loci, MLVA types matching at eight or more loci were regarded as clonally related. Consequently, eight distinct MLVA groups were established and the grouping feature established with the MLVA types had good agreement with that built with ST types (Figure 1). The two serogroup A isolates were characterized as different MLVA types (TW48 and TW59), differing in three loci, both carried ST-7 type within the ST-5 complex (Table 2). Similar to the results obtained from the previous MLST analysis, a complicated clonal relationship was found among the 52 serogroup B isolates. The majority of MLVA types were distributed in three major MLVA groups, T2, T3 and T4, and 13 types were regarded as single clonal lineages. T2 group comprised 10 MLVA types: isolates within this group and with TW10 (differing in five loci with the closest TW16 in T2 group) carried ST types belonging to the ST-3439 group. Similar to the T2 group, the T3 group comprised 11 MLVA types and all the isolates in this group belonged to the ST-3200 group. With the exception of TW39, isolates with the MLVA types within T4 group belonged to the ST-41/44 complex. In the MST analysis with ST types, some isolates were grouped in the ST-41/44 complex, but with MLVA allelic profiles they (TW1, TW2, TW27, TW55 and TW63) were separated from the T4 group. However, they had a closer genetic relationship with the genotypes within the T4 group.

All the MLVA types, except TW65 and TW88, representing the serogroup W135 isolates, were clustered in T7 group. The two MLVA types (TW25 and TW52), identified in three serogroup C isolates, had a closer clonal relationship with the W135 isolates than other serogroup isolates, although they differed at five loci with the closest MLVA types within the T7 group. A total of 32 MLVA types were identified in the 31 serogroup W135 and three serogroup C isolates; in contrast, only two ST types (ST-11 and its single locus variant, ST-3016) were found in the isolates (Table 2). The isolates with TW25 and TW52 types emerged in 2001 and 2002, respectively. Since TW25 and TW52 differed in as many as seven loci, the two MLVA strains should not be derived from a common imported strain.

The serogroup Y isolates shared a close clonal relationship as the seven MLVA types, forming a compact cluster. Six MLVA types differed in only one or two loci with the founder type, TW3, which was identified in the earliest collected isolates in Taiwan.

### MLVA allelic profiles of isolates from patient-contact episodes

Five isolates, collected from healthy contacts of four patients were characterized by MLVA. The MLVA profiles were identical for isolates from three episodes. Two isolates from the fourth episode differed in a single locus, NMTR-7 (Table 4).

## Discussion

Our data demonstrate that the MLVA method is powerful for subtyping and useful for phylogenetic investigation of *N. meningitidis *isolates. The MLVA exhibited a much higher discriminatory power than PFGE for the isolates tested and the resulting data agreed well with the epidemiological observations. Of the 100 *N. meningitidis *isolates characterized, 96 were collected from sporadic cases with no apparently epidemiological links. This MLVA method with 12 VNTR loci discriminated, not only all the genetically diversified serogroup B isolates, but also exhibited a high degree of resolution for the serogroup W135 isolates. Although serogroup W135 meningococci emerged in Taiwan before 1996, only four PFGE patterns, sharing a high pattern similarity, were identified in the 31 isolates collected from 1996 to 2002 [9]. However, MLVA differentiated the 31 isolates into 30 genotypes. MLVA data also supported the belief that serogroup Y meningococci were derived from a recently emerging clone in Taiwan. Serogroup Y meningococci emerged for the first time in 2001 in Taiwan; the 11 isolates collected in 2001 to 2002 were tightly clustered together. The clustering feature supported the observation that the serogroup Y clone was recently introduced into this country. MLVA genotyping also showed that there were no a major epidemic *N. meningitidis *strain circulating in the country, where meningococcal disease was infrequent.

Our study showed that the clonal relationships between the isolates, established with MLVA types, was in good agreement with those built with ST types. As shown on Figure 1, strains within a ST complex or ST group shared more common VNTR loci. Among the 12 loci, four (NMTR1, NMTR2, NMTR7 and NMTR 12) were highly polymorphic; they could have higher variation rates. The remaining loci could have moderate and low variation rate. Thus, different sets of VNTR loci may be useful for phylogenetic investigation of isolates evolving over different time scales. Phylogenetic investigations of spreading of *N. meningitidis *strains over a long time scale will best be carried out using loci with a low or moderate variation rate. Forensics and outbreak investigations may use loci with a higher variation rate. In our study, the MST grouping features built with 10 or 11 loci, which excluded one or two highly polymorphic loci, such as NMTR1, NMTR2 or both from 12 loci, remained similar but tighter to that with 12 loci (data not shown). Therefore, use of more VNTR loci with a lower variation rate will increase the power of MLVA in phylogenetic studies of *N. meningitidis *strains evolving over a long time scale.

The allelic profiles of the 11 serogroup Y isolates demonstrated the level of stability for the 12 VNTR loci. The comparison of the allelic profiles indicated that VNTR2 had the highest variation rate; five additional alleles at NMTR2, but only one at NMTR1, NMTR7 and NMTR9, evolved in the serogroup Y isolates over a 2-year time span. The stability of the VNTR loci was also demonstrated by the comparison of the allelic profiles of isolates from four patient-contact episodes. Although a single locus variant was observed in isolates from a patient-contact episode (Table 4), this MLVA method should be stable enough for forensic and outbreak investigations. Since variation normally occurs in only a small portion of isolates from an outbreak [15], such variation is usually not a problem for interpretation of MLVA data.

The MLVA is useful for identification of outbreak strains. In our record, there was no serogroup C meningococcus identified in 1996–2000. A serogroup C isolate (with TW25 genotype) was identified for the first time in 2001 and two (TW52 genotype) in 2002. Although the three isolates differed in PFGE patterns, they had the same ST-11 type [9]. Therefore, the 2002 isolates were considered deriving from the 2001 strain. However, TW25 and TW52 differed in seven loci, including the high polymorphic loci (NMTR1, NMTR2, NMTR7, and NMTR9) and three moderate polymorphic loci (NMTR12, NMTR18 and NMTR19). MLVA profiles suggested that the two MLVA strains should not derive from a common imported strain. In contrast with, MLVA results suggested that the serogroup Y isolates were evolved from a newly imported clone; strains derived from the clone still caused infections in 2003 to 2005 with 2–3 patients a year.

To date, in our study and that of Yazdankhah et al. [20], a total of 12 VNTR loci have been characterized, and more could still exist in the genomes. For example, Jordan et al. [22] identified 22 coding tandem repeat loci that varied in numbers of repeat units between the three sequenced strains, of which only five loci were included in this study. Although the rest of the loci may have lower allelic polymorphism, they may be included in the VNTR set suitable for phylogenetic investigation of *N. meningitidis *strains.

Of the 12 loci, five were not with repeat units of multiples of 3 bp. However, it is not necessary that a repeat unit needs to be of multiples of 3 bp for having a biological function. For example, NMTR1 locus, having a 7-bp repeat unit, is located within the coding sequence of the glycosyltransferase (PglE) gene that involves in pilin glycosylation and phase variation [23]. Tandem repeat sequences or repeat sequence tracts are usually involved in diverse biological functions; to date, more than 100 repeat associated phase-variable genes in *Neisseria *spp. have been identified [21,22,24]. Repeat loci may locate within the coding region of a gene or in the non-coding region involved in gene regulation [24]. The biological function of NMTR2, NMTR12 and NMTR19 has not been elucidated. NMTR12 is a compound repeat locus with 12- and 13-bp repeat units; the two repeat units arranged in variable numbers and sequences. Further investigation is needed to explore the biological functions associated with these repeat loci.

## Conclusion

MLVA exhibits a higher degree of resolution than PFGE for fine typing of *N. meningitidis *isolates and produces portable data that can easily be used for comparisons between laboratories via the Internet. MLVA data can also be used to investigate phylogenetic relationship between *N. meningitidis *strains. Therefore, MLVA can be adopted as an epidemiological tool for forensics and disease outbreak investigations, and for investigating clonal relationship among meningococcal strains. However, the mutation rate for each VNTR loci is still unknown. To fully exploit the value of MLVA, more VNTR loci need to be explored and more *N. meningitidis *isolates, of known epidemiological history, need to be characterized.

## Methods

### Identification of VNTR loci

The genomes of *N. meningitidis *strains Z2491 (GenBank accession no. AL157959), MC58 (GenBank accession no. AE002098) and FAM18 (obtained from The Welcome Trust Sanger Institute [25]), were explored for potential VNTR loci using unpublished VNTRDB computer software developed by Kao et al. in National Taiwan University. The program, which incorporates the algorithm of the Tandem Repeat Sequence Finder software [26], searches tandem repeat loci from one of the three genomic sequences and then locates the positions of each of the loci at the other two compared genomes. The three genomic sequences are used in turn as the "parent" sequence, so that a locus with only one repeat unit at a genome, but with two or more repeat units at other genomes, will not be missed. Searches found more than 300 repeat loci that were common to all the three strains and had variable repeat units between the three strains. Twenty-three repeat loci that had short repeat unit length (≤ 30 bp), more than 85% repeat sequence identity, and no indels were selected for further evaluation with 10 genetic distinct strains. Twelve loci, which were detected in all of the 10 testing isolates and amplified with only one amplicon, were chosen for genotyping of *N. meningitidis *isolates (Table 1).

### Preparation of crude bacterial DNA

Meningococcal isolates, stored at -70°C, were plated onto trytic soy agar with 5% sheep blood and incubated overnight at 37°C under a 5% CO_2 _atmosphere. A loopful (10 μl) of bacterial growth was removed from the plate, suspended in 100 μl of TE buffer (10 mM Tris-Cl, 1 mM EDTA, pH 8.0) in an Eppendorf tube, and boiled for 10 min. After centrifugation at 3700 *g *for 10 min, the supernatant was transferred to a new tube and used for PCR amplification.

### PCR amplification and analysis of VNTR regions

The primer sets specific to the 12 VNTR regions are listed on Table 5. The primers were designed using the free program available at the Primer3 website [27]. A primer of each primer set was labeled on 5' end with an ABI-compatible dye, 6-FAM, NED, VIC or PET by the manufacture (Applied BioSystems, Foster City, CA, USA). Each 10-μl PCR mixture contained 1 × PCR buffer, 1.5 mM MgCl_2_, 0.4 μM each primer, 200 μM each deoxyribonucleotide, 1.0 unit of the recombinant SuperNew Taq DNA polymerase (Jier Sheng Company, Taipei, Taiwan), and 1 μl of DNA template prepared as above-mentioned. The samples were placed on a GeneAmp PCR System 9600 (Applied BioSystems) and the PCR reaction was performed with a denaturing step at 94°C for 5 min, followed by 30 cycles of amplification step at 94°C for 30 s, at 54°C for 45 s, and at 72°C for 45 s, and by an extension step at 72°C for 10 min. Three microliters of each PCR products was electrophoresed in 2% SeaKem LE agarose (Cambrex Bio Science, Rockland, ME, USA) to check the sizes of amplified DNA products and the quality of PCR amplification.

Before size analysis the fluorescent amplicons were diluted in water, usually at a 1:100 or 1:200 ratio, then separated by capillary electrophoresis on an ABI Prism 3130 Genetic Analyzer with GeneScan 500 LIZ Size Standard (cat # 4322682; Applied BioSystems). Data were collected and lengths of amplicons were determined with GeneScan Data Analysis Software, ver 3.7 (Applied BioSystems). All amplicons with different lengths from each locus were subjected to nucleotide sequence determination to verify the repeat sequence and the numbers of repeat units in the amplicons. The primers (without dye label) used for nucleotide sequence determination were the same as the primer sets used for PCR amplification. DNA sequencing was performed using the ABI Prism Big Dye Terminator cycle sequencing ready reaction kit and an ABI Prism 3130 Genetic Analyzer. The numbers of repeat units for the 12 VNTR loci (Table 1) and the predicted sizes of amplicons (Table 5) for the *N. meningitidis *strains Z2491, MC58 and FAM18 were taken as the standards to infer the number of repeat unit of each locus for the isolates tested.

### Data analysis

The numbers of repeat units for each locus were saved as "Character Type" data in BioNumerics software (version 3.5; Applied Maths, Kortrijk, Belgium) and then subjected to cluster analysis using the Minimum Spanning Tree method. The polymorphism information index or Nei's diversity index (DI) was calculated for evaluating allele diversity as 1-Σ (allele frequency)^2^.

### Bacterial strains

A total of 105 *N. meningitidis *isolates, collected from meningitis patients and healthy contacts, were included in this study. The collection from patients comprised 2 serogroup A isolates, 52 serogroup B isolates, 3 serogroup C isolates, 31 serogroup W135 isolates, 11 serogroup Y and 1 non-groupable isolate (Table 2). They were collected from sporadic cases between 1996 and 2002 in Taiwan, except two pairs of isolates (NM255 and NM256; NM377 and NM378), which were, respectively, isolated from a meningococcal disease outbreak in a family and from two cases with a close temporal and spatial connection. All the 100 isolates from patients have been characterized by PFGE and MLST in a previous study by Chiou et al. [9]. Five isolates from healthy contacts were collected from four independent patient-contact episodes (Table 4).

## Authors' contributions

JC Liao designed all the primers and MLVA analyzed all the isolates. CC Li was in charge of searching potential VNTR loci by computer software and MST clustering analyses. CS Chiou initiated and managed the project, analyzed data, and wrote the report. All authors read and approved the final manuscript.

## Figures and Tables

**Table 1:** VNTR locus characteristics at genomes of *N. meningitidis *strains Z2491, MC58 and FAM18.

VNTR locus^a^	Consensus sequence(s) of repeat unit^b^	Length of repeat unit (bp)	Locus in Z2491		Locus in MC58		Locus in FAM18		Function (Reference or locus_tag^c^)
				
			Location	Number of repeat unit	Location	Number of repeat unit	Location	Number of repeat unit	
NMTR1 (VNTR01)	CAAACAA	7	814844–815018	25	657240 – 657484	35	601072 – 601274	29	glycosyl transferase [23]
NMTR2	CATTTCT	7	920757 – 920875	17	773274 – 773301	4	716022 – 716154	19	Unknown
NMTR6	GCTTCAGTTACAGCTTCTTTG	21	1603619 – 1603660	2	1518318 – 1518359	2	1407985 – 1408068	4	membrane protein (NMA1680)
NMTR7	CAAG	4	1638925 – 1638972	12	1556771 – 1556814	11	1444059 – 1444090	8	hypothetical protein (NMB1507)
NMTR9 (VNTR06 & VNTR08)	GCCAAAGTT	9	2158594 – 2158514	9	285906 – 285968	7	277433 – 277666	26	rotamase (NMA2206)
NMTR9a	CCGCTGCTACTGCCGCTGCTGAAGCACCTG	30	1100635 – 1100694	2	970825 – 970854	1	932818 – 932907	3	dihydrolipoamide succinyltransferase E2 component (NMA1150)
NMTR9b	TACGGCTGCCGCGTCAAA	18	1385171 – 1385206	2	1293181 – 1293216	2	1191565 – 1191582	1	murein hydrolase (NMA1488)
NMTR9c	CGGATACGCTCTTGG	15	1446130 – 1446174	3	1353481 – 1353510	2	1250095 – 1250139	3	hypothetical protein (NMA1547)
NMTR10	CAGATT	6	2058538 – 2058515	4	386427 – 386480	9	1824619 – 1824596	4	DNA-directed RNA polymerase-β-chain (NMA0141)
NMTR12 (VNTR02)	a:GGGCTGTAGAGAT b: GGCTGTAGAGAT	13, 12	1234098 – 1234135	3 = 2a1b	1131164 – 1311531	29 = 20a9b	1043723 – 1044023	24 = 13a11b	Unknown
NMTR18	GGGTAGCGG	9	2052950 – 2052967	2	392028 – 392045	2	1819003 – 1819047	5	aldose 1-epimerase (NMA2099)
NMTR19	CGTATTTTCCCAT	13	2075417 – 2075442	2	369378 – 369403	2	1844470 – 1844534	5	Unknown

^a ^Loci in parentheses have previouslybeen characterized by Yazdankhah et al.[20]

^b^NMTR12 is a compound tandem repeat locus with 12- and 13-bp repeat units, arranged in variable numbers and sequences.

^c^Locus tag in parentheses are based on gene annotation of *N. meningitidis *strain Z2491 (GenBank accession no. AL157959), except the NMTR7 locus, which is based on gene annotation of strain MC58 (GenBank accession no. AE002098).

**Table 2:** ST, PFGE and MLVA genotypes for 100 *N. meningitidis *isolates.

Strain code	Year of Isolation	Serogroup	ST code^a^	PFGE code^a^	MLVA code	MLVA allelic profile^b^ (NMTR1, 2, 6, 7, 9, 9a, 9b, 9c, 10, 12, 18, 19)
ST-5 complex/Subgroup III						
NM77	2001	A	ST-7	NMEN06.0065	TW59	27, 14, 2, 3, 6, 1, 2, 4, 4, 22, 2, 2
NM320	2002	A	ST-7	NMEN06.0066	TW48	21, 17, 2, 3, 6, 1, 2, 4, 4, 23, 2, 2
ST-11 complex/ET-37 complex						
MS4527	1996	W135	ST-11	NMEN06.0056	TW87	47, 8, 4, 11, 33, 2, 1, 3, 4, 6, 6, 5
NM6	1996	W135	ST-11	NMEN06.0056	TW76	37, 8, 4, 12, 37, 3, 1, 3, 4, 24, 6, 5
NM7	1996	W135	ST-11	NMEN06.0056	TW90	58, 8, 4, 17, 34, 2, 1, 3, 4, 6, 6, 5
NM19	1997	W135	ST-11	NMEN06.0056	TW92	33(34), 7, 4, 11, 33, 3, 1, 3, 4, 24, 6, 5
NM24	1998	W135	ST-11	NMEN06.0056	TW74	36, 7, 4, 15, 32, 3, 1, 3, 4, 24, 6, 5
2002-060	2001	W135	ST-11	NMEN06.0056	TW36	16, 7, 4, 10, 23, 3, 1, 3, 4, 24, 6, 5
NM163	2001	W135	ST-11	NMEN06.0056	TW93	41(42), 8, 4, 9, 34, 3, 1, 3, 4, 25, 6, 5
NM21125	2001	W135	ST-11	NMEN06.0056	TW77	37, 9, 4, 12, 32, 3, 1, 3, 4, 24, 6, 5
NM64	2001	W135	ST-11	NMEN06.0056	TW79	38, 6, 4, 12, 35, 3, 1, 3, 4, 24, 6, 5
NM66	2001	W135	ST-11	NMEN06.0056	TW58	27, 6, 4, 15, 39, 3, 1, 3, 4, 24, 6, 5
NM76	2001	W135	ST-11	NMEN06.0056	TW83	39, 8, 4, 18, 34, 3, 1, 3, 4, 25, 6, 5
NM79	2001	W135	ST-11	NMEN06.0056	TW72	35, 10, 4, 12, 32, 3, 1, 3, 4, 24, 6, 5
NM80	2001	W135	ST-11	NMEN06.0056	TW57	27, 5, 4, 10, 37, 3, 1, 3, 4, 24, 6, 5
2002-059	2002	W135	ST-11	NMEN06.0056	TW78	38, 5, 4, 12, 36, 3, 1, 3, 4, 16, 6, 5
NM181	2002	W135	ST-11	NMEN06.0056	TW81	38, 9, 4, 11, 29, 3, 1, 3, 4, 24, 6, 5
NM25845	2002	W135	ST-11	NMEN06.0056	TW84	42, 6, 4, 21, 34, 3, 1, 3, 4, 23, 6, 5
NM293	2002	W135	ST-11	NMEN06.0056	TW66	32, 9, 4, 12, 36, 3, 1, 3, 4, 24, 6, 5
NM321	2002	W135	ST-11	NMEN06.0056	TW67	34, 9, 4, 12, 36, 3, 1, 3, 4, 24, 6, 5
NM5	1996	W135	ST-11	NMEN06.0056	TW70	35, 6, 4, 10, 37, 3, 1, 3, 4, 24, 6, 5
NM12	1997	W135	ST-11	NMEN06.0057	TW80	38, 6, 4, 14, 37, 3, 1, 3, 4, 24, 6, 5
NM14	1997	W135	ST-11	NMEN06.0057	TW91	59, 7, 4, 15, 34, 2, 1, 3, 4, 6, 6, 5
NM4967	1997	W135	ST-11	NMEN06.0057	TW75	37, 6, 4, 15, 38, 3, 1, 3, 4, 24, 6, 5
NM19172	2000	W135	ST-11	NMEN06.0057	TW61	28, 5, 4, 13, 38, 3, 1, 3, 4, 24, 6, 5
NM102	2001	W135	ST-11	NMEN06.0057	TW14	10, 10, 4, 9, 19(27), 3, 1, 3, 4, 24, 6, 5
NM152	2001	W135	ST-11	NMEN06.0057	TW82	39, 5, 4, 16, 37, 3, 1, 3, 4, 24, 6, 5
NM357	2002	W135	ST-11	NMEN06.0057	TW65	30, 7, 5, 25, 35, 3, 1, 3, 4, 24, 7, 5
NM60	2001	W135	ST-11	NMEN06.0059	TW71	35, 8, 4, 27, 35, 3, 1, 3, 4, 25, 6, 6
NM68	2001	W135	ST-11	NMEN06.0059	TW86	43, 7, 4, 10(11), 25, 3, 1, 3, 4, 24, 3, 6
NM257	2002	W135	ST-11	NMEN06.0059	TW88	49, 6, 4, 13, 24, 3, 1, 3, 4, 24, 3(6), 7
NM81	2001	C	ST-11	NMEN06.0067	TW25	11, 5, 4, 16, 27, 3, 1, 2, 4, 21, 6, 4
NM377	2002	C	ST-11	NMEN06.0068	TW52	24, 6, 4, 13, 24, 3, 1, 2, 4, 18, 4, 5
NM378	2002	C	ST-11	NMEN06.0068	TW52	24, 6, 4, 13, 24, 3, 1, 2, 4, 18, 4, 5
NM25	1998	W135	ST-3016	NMEN06.0058	TW51	23, 7, 4, 9, 32, 2, 1, 3, 4, 6, 6, 5
NM18972	2000	W135	ST-3016	NMEN06.0058	TW51	23, 7, 4, 9, 32, 2, 1, 3, 4, 6, 6, 5
ST-23 complex/Cluster A3						
NM21468	2001	Y	ST-23	NMEN06.0060	TW3	4, 18, 5, 7, 9, 1, 2, 2, 11, 24, 2, 2
NM22034	2001	Y	ST-23	NMEN06.0060	TW3	4, 18, 5, 7, 9, 1, 2, 2, 11, 24, 2, 2
NM82	2001	Y	ST-23	NMEN06.0060	TW3	4, 18, 5, 7, 9, 1, 2, 2, 11, 24, 2, 2
NM25569	2002	Y	ST-23	NMEN06.0060	TW7	4, 18, 5, 6, 9, 1, 2, 2, 11, 24, 2, 2
NM267	2002	Y	ST-23	NMEN06.0060	TW5	4, 19, 5, 7, 9, 1, 2, 2, 11, 24, 2, 2
NM28225	2002	Y	ST-23	NMEN06.0060	TW5	4, 19, 5, 7, 9, 1, 2, 2, 11, 24, 2, 2
NM100	2001	Y	ST-23	NMEN06.0061	TW6	4, 20, 5, 7, 9, 1, 2, 2, 11, 24, 2, 2
NM153	2001	Y	ST-23	NMEN06.0061	TW8	4, 15, 5, 7, 10, 1, 2, 2, 11, 24, 2, 2
NM21519	2001	Y	ST-23	NMEN06.0061	TW4	4, 21, 5, 7, 9, 1, 2, 2, 11, 24, 2, 2
NM21675	2001	Y	ST-23	NMEN06.0061	TW4	4, 21, 5, 7, 9, 1, 2, 2, 11, 24, 2, 2
NM25238	2001	Y	ST-23	NMEN06.0061	TW11	5, 23, 5, 7, 9, 1, 2, 2, 11, 24, 2, 2
ST-32 complex/ET-5 complex						
NM159	2001	B	ST-3465	NMEN06.0042	TW39	17, 8, 2, 12, 12, 1, 2, 3, 9, 26, 2, 3
ST-41/44 complex/Lineage 3						
NM21700	2001	B	ST-41	NMEN06.0002	TW85	40, 4, 2, 18, 10, 1, 1, 3, 4, 3, 2, 3
NM25135	2001	B	ST-41	NMEN06.0002	TW89	58, 4, 2, 11, 10, 1, 1, 3, 4, 4, 2, 3
2002-075	2002	B	ST-41	NMEN06.0002	TW18	10, 4, 2, 8, 10, 1, 1, 3, 4, 4, 2, 3
NM30607	2002	B	ST-41	NMEN06.0002	TW73	36, 4, 2, 10, 13, 1, 1, 3, 4, 4, 3, 3
NM20	1997	B	ST-41	NMEN06.0003	TW23	11, 5, 2, 7, 12, 1, 1, 3, 4, 4, 2, 3
NM39	1999	B	ST-41	NMEN06.0004	TW37	16, 8, 2, 12, 12, 1, 1, 3, 4, 3, 2, 3
NM25614	2002	B	ST-41	NMEN06.0046	TW34	15, 6, 2, 7, 12, 1, 1, 3, 4, 4, 2, 4
NM295	2002	B	ST-41	NMEN06.0046	TW33	15, 6, 2, 8, 12, 1, 1, 3, 4, 4, 2, 4
NM9	1996	B	ST-41	NMEN06.0047	TW17	10, 5, 2, 7, 14, 1, 1, 3, 4, 4, 2, 3
NM84	2001	B	ST-41	NMEN06.0047	TW38	17, 7, 2, 10, 12, 1, 1, 3, 4, 4, 2, 4
NM40	1999	B	ST-41	NMEN06.0090	TW41	18, 5, 2, 15, 16, 1, 1, 3, 4, 4, 2, 3
NM21261	2001	B	ST-154	NMEN06.0001	TW31	14, 8, 2, 11, 10, 1, 1, 3, 4, 3, 2, 3
NM24481	2001	B	ST-154	NMEN06.0001	TW46	20, 9, 2, 11, 10, 1, 1, 3, 4, 3, 2, 3
NM8	1996	B	ST-437	NMEN06.0025	TW63	28, 15, 2, 11, 9, 1, 1, 3, 8, 4, 3, 2
NM18	1997	B	ST-437	NMEN06.0044	TW55	26, 13, 2, 9, 8, 1, 1, 3, 8, 4, 3, 2
NM32	1998	B	ST-3466	NMEN06.0031	TW27	12, 6, 5, 8, 3, 1, 1, 3, 8, 4, 3, 2
Hua443	2002	B	ST-3468	NMEN06.0048	TW2	0, 16, 3, 15, 16, 1, 3, 3, 10, 3, 3, 3
2002-061	2002	B	ST-3468	NMEN06.0049	TW1	0, 14, 3, 14, 16, 1, 3, 3, 9, 3, 3, 3
ST-162 complex						
NM420	2002	B	ST-162	NMEN06.0037	TW42	18, 7, 2, 8, 13, 1, 2, 3, 25, 10, 3, 2
ST-865 complex						
NM15	1997	B	ST-865	NMEN06.0029	TW64	29, 21, 2, 7, 22, 1, 1, 2, 10, 0, 2, 2
NM272	2002	B	ST-865	NMEN06.0030	TW56	26, 14, 2, 10, 21, 1, 1, 2, 11, 0, 2, 2
ST-3129 group						
NM2	1996	B	ST-3129	NMEN06.0020	TW40	17, 13, 2, 8, 34, 1, 1, 2, 11, 8, 3, 2
NM13	1997	B	ST-3129	NMEN06.0027	TW69	34, 12, 4, 8, 16, 1, 1, 2, 10, 7, 3, 2
ST-3200 group						
NM21435	2001	B	ST-3200	NMEN06.0005	TW53	25, 11, 3, 8, 16, 1, 1, 3, 8, 3, 2, 2
NM71	2001	B	ST-3200	NMEN06.0007	TW28	13, 11, 3, 10, 15, 2, 1, 3, 8, 3, 2, 2
NM30	1998	B	ST-3200	NMEN06.0010	TW50	22, 8, 3, 17, 15, 2, 1, 3, 8, 3, 2, 2
NM88	2001	B	ST-3200	NMEN06.0010	TW35	15, 13, 3, 13, 13, 2, 1, 3, 8, 3, 2, 2
NM397	2002	B	ST-3200	NMEN06.0010	TW49	22, 6, 3, 5, 12, 2, 1, 3, 8, 3, 2, 2
NM10	1996	B	ST-3200	NMEN06.0012	TW44	19, 8, 3, 13, 10, 2, 1, 3, 8, 3, 2, 2
NM255	2002	B	ST-3441	NMEN06.0010	TW62	28, 8, 3, 10, 16, 2, 1, 3, 8, 3, 2, 2
NM256	2002	B	ST-3441	NMEN06.0010	TW62	28, 8, 3, 10, 16, 2, 1, 3, 8, 3, 2, 2
NM21992	2001	B	ST-3469	NMEN06.0006	TW45	19, 7, 3, 9, 12, 2, 1, 3, 8, 3, 2, 2
NM30088	2002	B	ST-3470	NMEN06.0013	TW13	8, 8, 3, 10, 9, 2, 1, 3, 8, 3, 2, 2
NM390	2002	B	ST-3503	NMEN06.0014	TW21	10, 9, 3, 8, 9, 2, 1, 3, 8, 3, 2, 2
NM26447	2002	B	ST-4836	NMEN06.0010	TW68	34, 11, 3, 15, 13, 2, 1, 3, 8, 4, 2, 2
ST-3439 group						
NM62	2001	B	ST-1393	NMEN06.0093	TW10	5, 34, 3, 13, 17, 1, 1, 3, 14, 5, 3, 2
NM16	1997	B	ST-3192	NMEN06.0023	TW16	10, 22, 3, 13, 14, 1, 1, 3, 15, 4, 3, 2
NM30397	2002	B	ST-3192	NMEN06.0024	TW22	10, 24, 3, 7, 16, 1, 1, 3, 14, 4, 3, 2
NM383	2002	B	ST-3192	NMEN06.0024	TW19	10, 20, 3, 13, 16, 1, 1, 2, 14, 4, 3, 2
NM21	1997	B	ST-3192	NMEN06.0091	TW20	10, 16, 3, 14, 15, 1, 1, 3, 14, 4, 3, 2
NM22	1997	B	ST-3192	NMEN06.0092	TW24	11, 21, 3, 10, 14, 1, 1, 3, 14, 4, 3, 2
Nm15656	1999	B	ST-3439	NMEN06.0016	TW9	5, 20, 3, 7, 14, 1, 1, 3, 15, 4, 3, 2
NM38	1999	B	ST-3439	NMEN06.0017	TW30	14, 20, 3, 13, 15, 1, 1, 3, 15, 4, 3, 2
NM37	1999	B	ST-3439	NMEN06.0089	TW15	10, 17, 3, 10, 13, 1, 1, 3, 18, 4, 3, 2
NM4	1996	B	ST-3440	NMEN06.0022	TW29	13, 16, 3, 10, 13, 1, 1, 3, 16, 4, 3, 2
NM22208	2001	B	ST-3442	NMEN06.0015	TW12	5, 30, 3, 10, 19, 1, 1, 3, 19, 4, 3, 2
Single clonal lineage						
NM3	1996	B	ST-3175	NMEN06.0021	TW26	11, 15, 4, 7, 9, 1, 2, 3, 11, 31, 2, 2
NM28	1998	B	ST-3196	NMEN06.0036	TW54	26, 9, 5, 21, 24, 1, 1, 3, 33, 9, 3, 2
NM90	2001	NT	ST-3366	NMEN06.0069	TW32	14, 4, 2, 7, 6, 1, 1, 3, 16, 8, 3, 2
NM15252	1999	B	ST-3437	NMEN06.0019	TW47	20, 13, 2, 14, 7, 1, 1, 3, 8, 17, 2, 2
NM25660	2002	B	ST-3438	NMEN06.0018	TW43	18, 19, 2, 18, 12, 1, 2, 3, 16, 7, 2, 2
NM412	2002	B	ST-3504	NMEN06.0050	TW60	27, 20, 2, 15, 15, 4, 2, 2, 10, 0, 2, 2

^a^Characterized previously [9].

^b ^Number in parentheses indicates the second copy of the locus. The second allele indicated in the parentheses was ignored in the MST analysis.

**Table 3:** Features of selected VNTR loci observed in 100 *N. meningitidis *isolates.

Locus	Length of repeat unit (bp)	Size range of amplicon (bp)	Range of repeat unit^a^	Number of alleles^a^	Polymorphism index^b^
NMTR1	7	197–589	3–59	40	0.96
NMTR2	7	236–446	4–34	23	0.92
NMTR6	21	165–228	2–5	4	0.73
NMTR7	4	195–291	3–27	18	0.9
NMTR9	9	189–513	3–39	28	0.94
NMTR9a	30	188–278	1–4	4	0.6
NMTR9b	18	182–218	1–3	3	0.33
NMTR9c	15	187–217	2–4	3	0.35
NMTR10	6	221–395	4–33	12	0.71
NMTR12	13, 12	218–572	3–31	19	0.81
NMTR18	9	182–227	2–7	6	0.67
NMTR19	13	186–251	2–7	6	0.64

^a^Not including the unamplifiable allele at NMTR1 and NMTR12.

^b^Nei's diversity index (DI) = 1 - ∑ (allele frequency)^2^

**Table 4:** MLVA profiles of *Neisseria meningitidis *isolates from four patient/contact episodes.

Patient/Contact	Sex	Age	Strain code	Year	Serogroup	MLVA code	MLVA profile (NMTR1, 2, 6, 7, 9, 9a, 9b, 9c, 10, 12, 18, 19)											
Patient P1	M	0.3	NM153	2001	Y	TW8	4	15	5	7	10	1	2	2	11	24	2	2
Contact of P1	F	29.3	NM156	2001	Y	TW8	4	15	5	7	10	1	2	2	11	24	2	2
Patient P2	M	0.4	Hua443	2002	B	TW2	0	16	3	15	16	1	3	3	10	3	3	3
Contact of P2	F	38.3	Hua452	2002	B	TW2	0	16	3	15	16	1	3	3	10	3	3	3
Patient P3	M	5.3	NM30397	2002	B	TW22	10	24	3	7	16	1	1	3	14	4	3	2
Contact of P3	NA	6.5	NM30464	2002	B	TW22	10	24	3	7	16	1	1	3	14	4	3	2
Contact of P3	NA	34.3	NM30465	2002	B	TW22	10	24	3	7	16	1	1	3	14	4	3	2
Patient P4	F	2.7	NM25614	2002	B	TW34	15	6	2	7	12	1	1	3	4	4	2	4
Contact of P4	F	24.3	NM25618	2002	B	TW33	15	6	2	8	12	1	1	3	4	4	2	4

NA: Not available

**Table 5:** VNTR locus-specific primers and the predicted sizes of amplicons from *N. meningitidis *strains Z2491, MC58 and FAM18.

Locus	Primer designation	Primer sequence (5' → 3')^a^	Tm(°C)	Predicted size of amplicon (bp)		
				
				Z2491	MC58	FAM18
NMTR1	NMTR-1 F	6-FAM-GGGTCAAAAGACGGAAGTGA	54.9	351	421	379
	NMTR-1 R	AAAATCATCCGAATCAATAAAGAC	49.8			
NMTR2	NMTR-2 F	PET-GTGCGCCAGTAAGAAAATACAAT	53.9	327	236	341
	NMTR-2 R	TCAGAAAAGTTTTGCATTTTGAA	50.1			
NMTR6	NMTR-6 F	6-FAM-GCGGCATCTTTCATTTTGTC	52.8	165	165	207
	NMTR-6 R	CGAAGAAGCGAAAGACCAAG	53.9			
NMTR7	NMTR-7 F	CCATCCTTATCCGAATCTGAA	55	231	227	215
	NMTR-7 R	VIC-CTGAAACCCTGCCTGAAGAA	53.4			
NMTR9	NMTR-9 F	PET-GGAAAGAATGATGAAAATCAAAGC	51.3	243	225	396
	NMTR-9 R	CCGTCTGAAAAGCGGATACC	55.8			
NMTR9a	NMTR-9a F	GTTGTTGCCGACCAAGTTTT	54.4	218	188	248
	NMTR-9a R	6-FAM-GAACCTTGCAATGCGTTCAC	55.2			
NMTR9b	NMTR-9b F	CGACTTCATCGTCCACAAAA	53	200	200	182
	NMTR-9b R	VIC-GGCTTTGTCTGCCTGTACG	56.3			
NMTR9c	NMTR-9c F	GGAAATCTGCGCTTTCGTAG	54	202	187	202
	NMTR-9c R	NED-TCATGTCAGCAATTCCCTCA	54			
NMTR10	NMTR-10 F	NED-GGCATCGATGATGTGAAACA	53.3	221	251	221
	NMTR-10 R	GTGCTGAAGCACCAAGTGAA	55.9			
NMTR12	NMTR-12 F	CAAAGAGAGAGTGGAAGAACATCA	54.5	218	548	481
	NMTR-12 R	PET-AATGACGAAGAGTGGCAGGATT	56.6			
NMTR18	NMTR-18 F	AACGGAAAATTCCTGCACAA	53.1	182	182	209
	NMTR-18 R	VIC-CGTTTTCCGTGTTCCTGATT	53.4			
NMTR19	NMTR-19 F	NED-GACATATTGTGCGATGTCGAG	53.3	186	186	225
	NMTR-19 R	CGCCAACAGAAAAGAATACGA	53.6			

^a ^6-FAM, VIC, NED and PET are ABI compatible dyes (Applied BioSystems, Foster City, CA, USA).
